# PATIENT-DERIVED XENOGRAFTS AS A PRECLINICAL MODEL FOR BONE SARCOMAS

**DOI:** 10.1590/1413-785220182602186998

**Published:** 2018

**Authors:** WALTER MEOHAS, REGINA ALCANTARA GRANATO, JOÃO ANTONIO MATHEUS GUIMARÃES, RHAYRA BRAGA DIAS, ANNELIESE FORTUNA-COSTA, MARIA EUGENIA LEITE DUARTE

**Affiliations:** 1. Centro de Oncologia Ortopédica, Instituto Nacional de Traumatologia e Ortopedia, Rio de Janeiro, RJ, Brazil,; 2. Department of Pathology, Universidade Federal Fluminense, Niterói, RJ, Brazil,; 3. Research Division, Instituto Nacional de Traumatologia e Ortopedia, Rio de Janeiro, RJ, Brazil.

**Keywords:** Bone neoplasms. Sarcoma, experimental. Translational medical research, Neoplasias ósseas, Sarcoma experimental, Pesquisa médica translacional.

## Abstract

**Objective:**

The purpose of this study was to reproduce a mouse model of bone sarcomas for use in cancer research.

**Methods:**

A fresh sample of the tumor tissue was implanted subcutaneously into nude mice. When the patient-derived xenograft (PDX) reached a volume of 1500 mm^3^, it was harvested for re-implantation into additional mice. Histology was used to compare the morphological characteristics of different generations of sarcoma xenografts with the primary tumor.

**Results:**

Sixteen sarcoma tissue samples were engrafted into nude mice. Nine patients were diagnosed with osteosarcoma, two with chondrosarcoma, two with malignant peripheral nerve sheath tumor, one with synovial sarcoma, one with pleomorphic sarcoma, and one with Ewing’s sarcoma. PDX tumors were generated in 11 of the 16 implanted specimens (69% success rate in P1). Six P1 tumors grew sufficiently for transfer into additional mice, producing the P2 generation, and three P2 tumors established the P3 generation.

**Conclusion:**

PDX tumors generated from bone sarcomas were successfully established in immunodeficient mice and reproduced the characteristics of the primary tumor with a high degree of fidelity. The preclinical PDX model described herein may represent an important tool for translational oncology research and for evaluating therapeutic strategies for bone sarcomas. Level of Evidence I; Experimental study.

## RESUMO

**Objetivo:**

O propósito deste estudo foi reproduzir em camundongos um modelo de sarcomas ósseos para uso em pesquisa oncológica.

**Método:**

Amostras frescas de tecido tumoral foram implantadas por via subcutânea em camundongos Nude. Quando o xenoenxerto derivado do paciente (PDX) alcançava 1500 mm^3^, ele era retirado do animal e reimplantado em outros camundongos. Estudos histológicos foram realizados para comparar as características morfológicas de diferentes gerações de xenoenxertos com o tumor primário.

**Resultados:**

Dezesseis amostras de tecido sarcomatoso foram enxertadas em camundongos. Nove pacientes foram diagnosticados com osteossarcoma, dois com condrossarcoma, dois com tumor maligno de bainha de nervo periférico, um com sarcoma sinovial, um com sarcoma pleomórfico e um com sarcoma de Ewing. Foram gerados tumores PDX em 11 das 16 amostras enxertadas (taxa de sucesso de 69% em P1). Destes, seis tumores P1 cresceram o suficiente para serem transferidos para outros camundongos, dando origem à geração P2 e três dos tumores P2 estabeleceram a geração P3.

**Conclusões:**

Os tumores PDX de sarcomas ósseos foram estabelecidos com sucesso em camundongos imunodeficientes e reproduziram com alta precisão as características do tumor primário. O modelo pré-clínico de PDX descrito pode representar uma ferramenta importante para a pesquisa oncológica translacional e para avaliar estratégias terapêuticas para sarcomas ósseos. Nível de Evidência I; Estudo experimental.

## INTRODUCTION

Bone sarcomas are a heterogeneous group of rare highly malignant tumors of unknown origin that constitute 0.2% of all adult malignancies and 5% of malignant neoplasms of children and young people.^1^ The clinical presentation of bone sarcomas is variable and nonspecific. In general, the symptoms of pain, swelling and functional loss are restricted to the lesion region. The pain can be intermittent, persistent, and progressive with irradiation. Another characteristic of bone sarcomas is the rapid local volume increase associated with altered skin coloration plus development of collateral circulation. Systemic symptoms such as fever, tiredness and weight loss are almost always associated with the metastatic spread of the disease.^2^

The classification of bone sarcomas is based on the cell type and the characteristics of the matrix produced by the tumor cells, which recapitulate the architecture of the original tissue. Most tumors differentiate into cell lines or tissues that make up the musculoskeletal system.^1^ Osteosarcoma is the most frequent primary bone sarcoma (35%), followed by chondrosarcoma (25%) and Ewing’s sarcoma (16%).^3^ Preclinical studies are designed to increase the knowledge about the biology of malignancies, the development of new therapeutic strategies and their validation before the phase of clinical trials. Although several *in vitro* and *in vivo* preclinical models are being widely used for decades to study cancer biology, the high rates of phase III clinical trials failures indicate that these preclinical models may have poor clinical predictive power.^4^ ^-^ ^7^

Animal models are currently an important tool for preclinical studies aiming to increase the knowledge about the biology of malignancies, the development of new therapeutic strategies and their validation before the phase of clinical trials. In order to translate results obtained in animal models into the clinical scenario, it is necessary that the animal model accurately reproduce the natural history of the disease. As the etiology and pathogenesis of sarcomas are unknown, the development of tumor models in general is incomplete and poorly reproducible. ^8^

Patient-derived tumor xenografts models, consisting on the implantation of fresh samples of patient’s tumor into immunodeficient mice are increasingly being used in cancer research as a basis for the development of new therapeutic strategies. Although tumor generated from xenografts are being reproduced in a murine immunodeficient environment, they recapitulate the biology of the original tumor and are easy to handle and maintain.^9^ ^,^ ^10^ Moreover, in these models it is possible to observe the development of metastases which allows to evaluate the behavior of the primary tumor mass and the secondary implant generated from the same tumor graft. Another important advantage of xenografts models is that human neoplastic cells proliferate in their native environment, maintaining the pattern of individual heterogeneity of each patient. Thus, from the tumor tissue implanted in the mice, it is possible to reproduce the pattern and biology of human tumor growth.^11^ ^-^ ^13^ Herein, we aimed to reproduce a mouse model of bone sarcomas for use in cancer research. This preclinical model will constitute an important platform for studies of sarcomas biology and may be used on therapeutic strategies as a prelude to subsequent translation to patients. 

## MATERIALS AND METHODS

This study was reviewed and approved by the Institutional Review Board (IRB) of the National Institute of Traumatology and Orthopedics (CAAE: 69859417.2.0000.5273), and all patients provided written informed consent as part of the above-mentioned IRB-approved protocol. Tumor tissue samples from patients with clinical diagnosis of a primary bone sarcoma were obtained at the time of biopsy or surgical resection from January 2017 to September 2017. Patients who were treated preoperatively with neoadjuvant chemoteraphy were also included in the study. The following clinical parameters were collect from all patients: age, gender, tumor location, histopathological diagnosis of the primary lesion and history of preoperative chemotherapy.

### Tumor samples

A fresh sample of the patient tumor was obtained from a representative biopsy or from the surgical specimen resulting from resection or limb amputation. For tumor samples obtained from surgical resection, the fragments were collected from solid areas, avoiding areas of necrosis and hemorrhage and maintained in DMEM medium at 4^o^C until implantation. Tumor tissue samples were cut into 3 x 3 x 3 mm^3^ pieces and a total of four pieces/animal was xenotransplanted. A single 2 x 5 mm cylindrical fragment of tissue obtained by incisional or fluoroscopy-guided needle biopsy represented samples from biopsies. Similar to the surgical specimens, tumor tissue was cut into 3 x 3 x 3 mm^3^ pieces. Tumor samples were collected, handled and stored under sterile conditions.

- Establishment of patient-derived xenograft (PDX tumor model)

**Figure 1:**
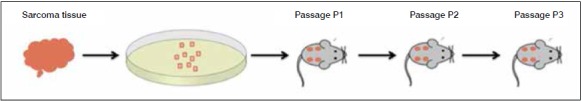
Methods for developing patient-derived xenograft model (PDX). Immunodeficient mice received human bone sarcoma tissue fragments (four pieces/animal) engrafted into the subcutaneous space of the flank.

Six to eight-week-old athymic nude mice (B6.Cg-Foxn1^nu^) were used in this study. All animal studies were approved by the Institutional Animal Care and Use Committee of the National Institute of Traumatology and Orthopedics (Protocol n^o^ 005/2017). During the entire experimental period the animals were kept in a barrier facility on a high efficiency particulate arrestance (HEPA)-filtered rack and were fed ad libitum with autoclaved laboratory rodent diet. For all surgical experiments the mice were anesthetized with isoflurane inhalation (2,5-5,0 Vol% per liter oxygen). Under sterile conditions each tumor fragment was implanted into a subcutaneous area on the right and left flanks. Tumor dimensions were checked twice weekly using a digital caliper and tumor volume (TV) was calculated according to the formula: TV= (length x width^2^)/2. When tumor size in the implanted area reached an approximate volume of 1500 mm^3^ they were harvested (P1 generation) for transplantation to the next generation into additional mice (P2, P3) (Figure 1). Conventional radiography images were acquired at the end of the experiment when the animals were euthanized by an overdose of ketamine and xylazine. Growth failure was considered in the absence of tumor growth four months after implantation.

- Histopathological characterization of primary and patient-derived bone sarcoma xenograft (PDX)

Fresh xenograft samples were fixed in 10% buffered formalin and paraffin-embedded blocks were prepared for all tumors. Sections (4mm thick) were stained with hematoxylin-eosin (H&E) for histological comparisons between the patient tumor and xenografts. 

## RESULTS

In total, sixteen surgically removed sarcoma tissues from fifteen patients (eleven male and four female aged 10-51 years old) were engrafted into nude mice. All tumor samples were obtained from primary sites. Eight patients were diagnosed with osteosarcoma, two with chondrosarcoma, two with malignant peripheral nerve sheath tumor (MPNST), one with synovial sarcoma, one with pleomorphic sarcoma (malignant fibrous histiocytoma) and one with Ewing sarcoma. Patient demographics and clinicopathologic characteristics for the harvested tumors are summarized in Table 1. 

**Table 1:** Clinical characteristics of donor patients used for generation of patient-derived xenografts (PDX).

Case No.	Age	Gender	Sarcoma histology	Location	Previous chemotherapy	Metastasis
1	23	Male	Osteosarcoma (telangiectatic)	Femur	No	Yes (lund and CNS)
2	11	Male	Osteosarcoma (central)	Femur	No	No
3	43	Male	Osteosarcoma (central)	Tibia	No	No
4	11	Male	Osteosarcoma (central)	Femur	Yes	No
5	17	Male	Osteosarcoma (central)	Femur	No	No
6	14	Female	Osteosarcoma (central)	Femur	Yes	Yes (lung)
7	13	Female	Osteosarcoma (central)	Fibula	Yes	No
8	14	Male	Osteosarcoma (central)	Femur	No	Yes (lung)
9	13	Female	Osteosarcoma (central)	Fibula	No	No
10	39	Male	Synovial sarcoma	Knee	Yes	No
11	23	Male	Grade I chondrosarcoma	Femur	No	No
12	40	Male	Grade II chondrosarcoma	Scapula	No	No
13	29	Male	Malignant peripheral nerve sheath tumor (MPNST)	Forearm	No	Yes (lung)
14	51	Female	Malignant peripheral nerve sheath tumor (MPNST)	Fibula	No	Yes (lung)
15	47	Male	Pleomorphic sarcoma (Malignant Fibrous Histiocytoma)	Knee	No	No
16	10	Male	Ewing sarcoma	Femur	Yes	No

Efficacy of establishment of primary patient-derived (PDX) bone sarcoma mouse xenograft

**Figure 2:**
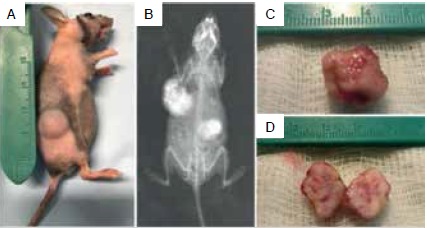
Bone sarcoma PDX. (A) First generation (passage 1) tumor growth six weeks after transplantation of patient-derived osteosarcoma into the subcutaneous of nude mice. (B) Representative X-ray image from PDX shown in (A). (C and D) Gross pathologic examination of first generation PDX displaying a bosselated 1,5 cm mass. (D) Cut surface has a fish flesh-like appearance characteristic of sarcomas, with focal areas of hemorrhage (pink).

Only fresh tumor samples obtained on the day of the surgical removal were implanted into nude mice (Figure 2). PDX tumors were initially generated (P1) in 11 out of 16 implanted specimens. Six of P1 tumors grew sufficiently for transfer into further mice giving rise to P2 generation and three of P2 tumors established the P3 generation. Sarcoma tissue from five patients (two osteosarcomas, one grade I chondrosarcoma, one grade II chondrosarcoma and one with Ewing sarcoma) failed to engraft into the mice.

Elapsed time for engraftment in P1 animals was 59 days (19-125 days), 28 days in P2 (18-40 days) and 18 days in P3 (16-21 days). Table 2 summarizes successful engraftment cases and growth rates.

**Table 2:** Characteristics of patient-derived xenografts (PDX) models for bone sarcomas.

Case No.	Histopathological diagnosis	Tumor sample source	Time for subcutaneous patient-derived xenograft (PDX) growth (days)		
			Passage 1	Passage 2	Passage 3
1	Osteosarcoma	Hip disarticulation	45	18	-
2	Osteosarcoma	Core needle biopsy	57	41	21
3	Osteosarcoma	Leg amputation	32	18	-
4	Osteosarcoma	Tumor ressection	48	29	-
5	Osteosarcoma	Core needle biopsy	125	-	-
6	Osteosarcoma	Tumor ressection	23	-	-
7	Osteosarcoma	Leg amputation	21	-	-
8	Osteosarcoma	Core needle biopsy	Failed	-	-
9	Osteosarcoma	Core needle biopsy	Failed	-	-
10	Synovial sarcoma	Hip disarticulation	19	-	-
11	Grade I chondrosarcoma	Tumor ressection	Failed	-	-
12	Grade II chondrosarcoma	Tumor ressection	Failed	-	-
13	Malignant peripheral nerve sheath tumor (MPNST)	Arm amputation	84	36	17
14	Malignant peripheral nerve sheath tumor (MPNST)	Core needle biopsy	64	30	17
15	Pleomorphic sarcoma (Malignant Fibrous Histiocytoma)	Tumor ressection	66	-	-
16	Ewing sarcoma	Tumor ressection	Failed	-	-

Histology of original patient tumor and PDX tumor

**Figure 3:**
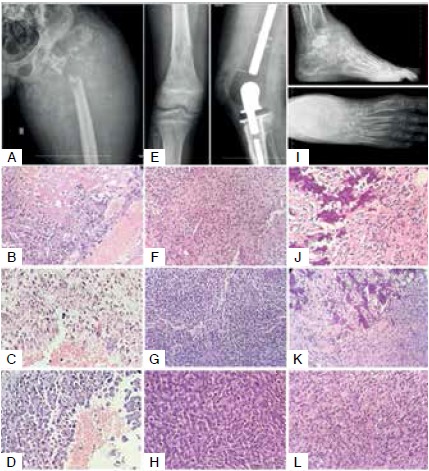
Radiographic and histologic features from primary and PDX tumors. (A) Telangiectatic osteosarcoma (OS) in a 23-year-old man (case 1). Radiograph of the proximal femur reveals a lytic lesion with extensive bone destruction and expansion into soft tissue. (B) Representative histology of the original patient tumor obtained by core needle biopsy and (C) during hip disarticulation. Microscopically telangiectatic OS is characterized by spaces containing blood (arrow head), with septa composed of malignant osteoblasts in a background of hypercellular and anaplastic stroma and tumor osteoid (*). (D) Blood-filled space lined by highly malignant osteoblasts in the xenograft tumor (P1) closely resembles the primary tumor. (E) Central OS in an 11-year-old boy (case 2). A diffuse lytic/blastic permeating lesion is seen in the lower femur with cortical destruction and tumor expansion into soft tissue. Limb-salvage surgery with endoprosthesis reconstruction after en-block tumor resection. (F) Malignant spindle or oval-spindle shape cell tumor with hyperchromatic nuclei surrounded by a small amount of cytoplasm. The production of neoplastic bone/osteoid is sparse. (G-H) The histology of primary OS characterized by the proliferation of spindly, oval or round neoplastic cells was retained in both P1 (G) and P2 (H) xenograft tumors. (I) Conventional OS in a 43-year-old man (case 3). Plain radiograph of ankle and foot showing a large ill-defined lytic lesion involving the talus and surrounding soft tissue. (J) Poorly formed neoplastic bone trabeculae are seen in the primary tumor in association with anaplastic cells displaying nuclear pleomorphism. (K-L) The xenograft tumor (P1) closely resembles the primary tumor including production of neoplastic bone, anaplasia and abundant mitotic figures (L). OS=Osteosarcoma. P1= PDX tumor in first passage; P2= PDX tumor in the second passage. H&E-stained sections. (B, F, G, J-L): Magnification x10; (C, D, H): Magnification x20.

**Figure 4:**
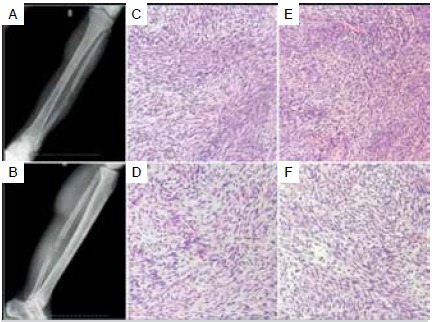
Radiographic and histologic features from MPNST primary tumor and PDX tumors. (A-B) MPNST in a 51-year-old woman (case 14). Large soft tissue mass overlying the fibula associated with extrinsic cortical erosion and bone destruction. (C) Primary MPNST characterized by neoplastic fusiform or spindle shaped cells arranged in dense cellular fascicles. (D-F) Histological features of corresponding patient tumor such as cell morphology, and the swirling cellular arrangement were largely reproduced in mouse passage P1 (D), P2 (E) and P3 (F) PDX tumors. MPNST= Malignant Peripheral Nerve Sheath Tumors. P1= PDX tumor in first passage; P2= PDX tumor in the second passage. P3= PDX tumor in the third passage. H&E-stained sections. (C-F): Magnification x10.

Microscopic examination of hematoxylin and eosin (H&E) stained histological sections demonstrated that the original tumor characteristics were preserved in the PDX. Mouse xenografts displayed strong histological similarity with the clinical specimens including tumor cellularity, tumor cell anaplasia, mitotic figures and formation of neoplastic extracellular bone matrix. Subgrafted xenograft tumors also retained the histopathological features of the original patient tumors indicating the same pattern of differentiation capacity (Figure 3 and 4).

## DISCUSSION

In contrast to the frequency of adenocarcinomas, bone sarcomas are rare, and for this reason, preclinical models such as PDX are more restricted and difficult to develop.^14^ On the other hand, it is well established that preclinical models is the key element in translational research with an obvious potential to promote significant advances in the area of cancer research, particularly for the development of new anti-neoplastic drugs.^8^

The process of generating xenograft models from primary or metastatic malignancies is already widely described in the literature.^15^ This increasing interest in PDX derives in large part from the potential of the model to preserve tumor growth kinetics, potential for local invasion and the ability to metastasize, and to reproduce the response to polychemotherapy treatment. Another frequently addressed issue in the literature concerns the site of tumor implantation - heterotopic or orthotopic - for the PDX generation. The orthoxenografts have the advantage of developing in the same anatomical microenvironment of the patient original tumor. For bone tumors, orthotopic implants would provide also better quality of imaging documentation.^16^ However, in the particular case of bone tissue, for an orthotopic tumor implantation it is necessary a complex surgical procedure that is greatly hampered by the delicate murine bone anatomy. 

The method described in this paper, to generate PDX models in mice from fresh bone sarcomas has several advantages: it is simple to perform, does not require complicated operative procedures, has high engraftment rates and the mouse-to-mouse subgrafting retain the morphological characteristics of the original grafted tumor. Of the 16 tumors that were implanted subcutaneously into the flanks of nude mice, 69% (11/16) successfully engrafted. Moreover, 54% (6/11) of P1 tumors were re-implanted into other animals and the remaining P1 tumors have not yet reached the established volume to be re-implanted. The morphology of the lesions developed in the PDX of different passages reproduced with high degree of fidelity the characteristics of the primary tumor. The retention of histological features, including cellular morphology and arrangement, histological subtype of corresponding sarcoma, and tissue architecture indicate that PDX tumors maintain the same pattern of differentiation of the original patient tumors. The phenotypic stability of PDX models was also confirmed in studies that demonstrated stable response rates to drug treatments up to 10 passages.^17^

In the present study we observed that a potential limiting factor to produce PDX tumors is a limited supply of tissue to develop the model, when the material is obtained from small tumor samples such as biopsy specimens. Also, in samples obtained from patients with excessively large tumors with extensive areas of necrosis and in post-chemotherapy samples the possibility of obtaining lower engraftment efficiency should be taken into account. Another important challenge for bone tumors is the technical difficulty to reproduce orthotopic PDX models and to ensure high tumor take rates. However, considering the high degree of biological aggressiveness of sarcomas, it is possible that tumor take rates do not constitute a limitation for bone sarcomas in particular. Indeed, studies with human breast cancer demonstrated that more aggressive tumors had a higher take rate.^11^ We believe that the cases that failed to engraft (5/16) were due to intrinsic tumor characteristics such as the relatively low biological aggressiveness of the sarcoma subtype (grade I and grade II chondrosarcoma), to the small sample size obtained by core needle biopsy, limiting the size of the implant (n=2) and the reduced potential for engraft due to necrosis, in one sample from a post-chemotherapy Ewing sarcoma. 

As the PDX recapitulates the biology of the human tumor they are predictive of clinical outcome, and consist in an important tool for the development of a personalized treatment. These advances in the area of translational cancer research led the US National Cancer Institute (NCI) to consider the replacement of tumor conventional cell line repositories for PDX samples due to the high similarity of the PDX models with the natural history and clinical outcome of pediatric bone cancer.^18^

## CONCLUSION

PDX tumors generated from bone sarcomas samples were successfully established in immunodeficient mice. The morphological similarities of PDX to the corresponding primary tumor confirm that the preclinical PDX model can be translated to clinical practice to stimulate the development of personalized approaches for the treatment of several types of bone sarcomas.
